# Supplementary material for: Effect of Dietary Patterns on Inflammatory Bowel Disease: A Machine Learning Bibliometric and Visualization Analysis
Source: Nutrients. 2023 Aug 3;15(15):3442. doi: 10.3390/nu15153442 (PMC10420952; doi:10.3390/nu15153442)
Supplement: Supplementary file 1 [file nutrients-15-03442-s001.zip › Supplementary Table S1.pdf]

Supplementary Table S1. Top 10 authors of related literature based on bibliometrix package

Rank	**Authors**	**Number of publications**
1	GHOSH S	10
2	ANANTHAKRISHNAN AN	8
3	CHAN AT	8
4	GASBARRINI A	8
5	HALLER D	8
6	COLOMBEL JF	7
7	DIJKSTRA G	7
8	GIBSON PR	7
9	NG SC	7
10	RAMAN M	7
11	SZILAGYI A	7
