# Supplementary material for: Effect of Dietary Patterns on Inflammatory Bowel Disease: A Machine Learning Bibliometric and Visualization Analysis
Source: Nutrients. 2023 Aug 3;15(15):3442. doi: 10.3390/nu15153442 (PMC10420952; doi:10.3390/nu15153442)
Supplement: Supplementary file 1 [file nutrients-15-03442-s001.zip › Supplementary Table S2.pdf]

Supplementary Table S2. Top 10 total citations or average citations based on OALM

**Rank**	**Institutions**	**Total citations**	**Institutions**	**Average citations**
1	Harvard Univ	397	Univ Paris 11	54.5
2	Univ Montreal	277	Hosp Univ Penn	53
3	Univ Penn	251	Umea Univ	52
4	Massachusetts Gen Hosp	218	Natl Inst Publ Hlth & Environm RIVM	52
5	Univ Calif San Francisco	180	Univ Med Ctr	52
6	Univ N Carolina	168	Malmo Univ Hosp	52
7	Karolinska Inst	134	DKFZ German Canc Res Ctr Heidelberg	52
8	Univ Calgary	118	Canc Res & Prevent Inst ISPO	52
9	Univ Manitoba	116	St Justine Univ Hosp	52
10	Harvard Med Sch	107	Nancy Univ Hosp	48
