# Supplementary material for: Effect of Dietary Patterns on Inflammatory Bowel Disease: A Machine Learning Bibliometric and Visualization Analysis
Source: Nutrients. 2023 Aug 3;15(15):3442. doi: 10.3390/nu15153442 (PMC10420952; doi:10.3390/nu15153442)
Supplement: Supplementary file 1 [file nutrients-15-03442-s001.zip › Supplementary Table S3.pdf]

Supplementary Table S3. Top 10 journals under multiple influence indicators based on bibliometrix package

Rank	Journals	h_index	Journals	g_index	Journals	Total citation
1	NUTRIENTS	22	NUTRIENTS	44	NATURE	7552
2	WORLD JOURNAL OF GASTROENTEROLOGY	16	INFLAMMATORY BOWEL DISEASES	26	GASTROENTEROLOGY	2136
3	INFLAMMATORY BOWEL DISEASES	15	WORLD JOURNAL OF GASTROENTEROLOGY	23	JOURNAL OF CLINICAL GASTROENTEROLOGY	2109
4	JOURNAL OF CROHNS & COLITIS	15	PLOS ONE	23	INTERNATIONAL JOURNAL OF MOLECULAR SCIENCES	2055
5	PLOS ONE	14	JOURNAL OF CROHNS & COLITIS	22	NUTRIENTS	2039
6	GASTROENTEROLOGY	14	INTERNATIONAL JOURNAL OF MOLECULAR SCIENCES	19	BRITISH JOURNAL OF NUTRITION	1321
7	INTERNATIONAL JOURNAL OF MOLECULAR SCIENCES	13	DIGESTIVE DISEASES AND SCIENCES	19	AMERICAN JOURNAL OF GASTROENTEROLOGY	1314
8	AMERICAN JOURNAL OF GASTROENTEROLOGY	11	GASTROENTEROLOGY	15	MICROORGANISMS	1145
9	FRONTIERS IN IMMUNOLOGY	10	FRONTIERS IN IMMUNOLOGY	15	EUROPEAN JOURNAL OF NUTRITION	1111
10	JOURNAL OF PEDIATRIC GASTROENTEROLOGY AND NUTRITION	10	JOURNAL OF PEDIATRIC GASTROENTEROLOGY AND NUTRITION	14	INFLAMMATORY BOWEL DISEASES	1098
10	DIGESTIVE DISEASES AND SCIENCES	10				
