# Supplementary material for: Effect of Dietary Patterns on Inflammatory Bowel Disease: A Machine Learning Bibliometric and Visualization Analysis
Source: Nutrients. 2023 Aug 3;15(15):3442. doi: 10.3390/nu15153442 (PMC10420952; doi:10.3390/nu15153442)
Supplement: Supplementary file 1 [file nutrients-15-03442-s001.zip › Supplementary Table S4.pdf]

Supplementary Table S4. Top 10 journals in the number of cited articles

Rank	Journals	The number of articles cited	IF	JCR Partition
1	GASTROENTEROLOGY	3351	33.883	1
2	GUT	3058	31.793	1
3	INFLAMM BOWEL DIS	2291	7.290	1
4	NATURE	1778	69.504	1
5	AM J GASTROENTEROL	1547	12.045	1
6	PLOS ONE	1345	3.752	2
7	P NATL ACAD SCI USA	1209	12.779	1
8	SCIENCE	1022	63.714	1
9	ALIMENT PHARM THER	954	9.524	
10	NUTRIENTS	925	6.706	1
