# Supplementary material for: Effect of Dietary Patterns on Inflammatory Bowel Disease: A Machine Learning Bibliometric and Visualization Analysis
Source: Nutrients. 2023 Aug 3;15(15):3442. doi: 10.3390/nu15153442 (PMC10420952; doi:10.3390/nu15153442)
Supplement: Supplementary file 1 [file nutrients-15-03442-s001.zip › Supplementary Table S5.pdf]

Supplementary Table S5. Top 15 references

Rank	Cited References	Citations	Year	Journal	IF	JCR Partition
1	TURNBAUGH PJ, 2006, NATURE	96	2011	SCIENCE	63.714	Q1
2	DEVKOTA S, 2012, NATURE	87	2011	GASTROENTEROL	12.045	Q1
3	ANANTHAKRISHNAN AN, 2014, GUT	84	2014	NATURE	69.504	Q1
4	RACINE A, 2016, INFLAMM BOWEL DIS	77	2012	GASTROENTEROLOGY	33.883	Q1
5	CHASSAING B, 2015, NATURE	74	2013	GASTROENTEROLOGY	33.883	Q1
6	COHEN AB, 2013, DIGEST DIS SCI	72	2010	Proc Natl Acad Sci U S A	12.779	Q1
7	JANTCHOU P, 2010, AM J GASTROENTEROL	69	2017	Lancet	202.731	Q1
8	JOWETT SL, 2004, GUT	61	2004	GUT	31.734	Q1
9	NG SC, 2017, LANCET	57	2010	GASTROENTEROL	12.045	Q1
10	DE FILIPPO C, 2010, P NATL ACAD SCI USA	54	2015	NATURE	69.504	Q1
11	ANANTHAKRISHNAN AN, 2013, GASTROENTEROLOGY	54	2013	Dig Dis Sci	3.487	Q3
12	MOLODECKY NA, 2012, GASTROENTEROLOGY	53	2014	GUT	31.734	Q1
13	DAVID LA, 2014, NATURE	53	2016	Inflamm Bowel Dis	7.290	Q1
14	HOU JK, 2011, AM J GASTROENTEROL	52	2012	NATURE	69.504	Q1

15	WU GD, 2011, SCIENCE	51	2006	NATURE	69.504	Q1
