# Supplementary material for: Effect of Dietary Patterns on Inflammatory Bowel Disease: A Machine Learning Bibliometric and Visualization Analysis
Source: Nutrients. 2023 Aug 3;15(15):3442. doi: 10.3390/nu15153442 (PMC10420952; doi:10.3390/nu15153442)
Supplement: Supplementary file 1 [file nutrients-15-03442-s001.zip › Supplementary Table S6.pdf]

Supplementary Table S6. Top 10 references of centrality and burst

**Rank**	**Centrality**	**References**	**Burst**	**References**
1	0.15	Frank DN, 2007, P NATL ACAD SCI USA	17.55	David LA, 2014, NATURE
2	0.09	Galvez J, 2005, MOL NUTR FOOD RES	15.70	Wu GD, 2011, SCIENCE
3	0.09	Wen L, 2008, NATURE	13.58	Ng SC, 2017, LANCET
4	0.08	Wu GD, 2011, SCIENCE	12.95	Molodecky NA, 2012, GASTROENTEROLOGY
5	0.08	Sokol H, 2008, P NATL ACAD SCI USA	12.24	Hou JK, 2011, AM J GASTROENTEROL
6	0.07	David LA, 2014, NATURE	12.02	Jostins L, 2012, NATURE
7	0.06	Gevers D, 2014, CELL HOST MICROBE	11.89	Levine A, 2019, GASTROENTEROLOGY
8	0.06	Agus A, 2016, SCI REP-UK	11.70	Levine A,2020, CLIN GASTROENTEROL H
9	0.06	Backhed F, 2005, SCIENCE	11.55	Yatsunenko T, 2012, NATURE
10	0.06	Lepage P, 2011, GASTROENTEROLOGY	11.23	Gevers D, 2014, CELL HOST MICROBE
