# Supplementary material for: Effect of Dietary Patterns on Inflammatory Bowel Disease: A Machine Learning Bibliometric and Visualization Analysis
Source: Nutrients. 2023 Aug 3;15(15):3442. doi: 10.3390/nu15153442 (PMC10420952; doi:10.3390/nu15153442)
Supplement: Supplementary file 1 [file nutrients-15-03442-s001.zip › Supplementary Table S7.pdf]

Supplementary Table S7. Top 15 Keywords based on CiteSpace 's degree, Centrality, Sigma

Rank	Centrality	Keywords	Degree	Keywords	Sigma	Keywords
1	0.35	inflammatory bowel disease	244	inflammatory bowel disease	1.68	ulcerative coliti
2	0.2	crohns disease	198	crohns disease	1.43	nf kappa b
3	0.2	ulcerative coliti	181	ulcerative coliti	1.36	colorectal cancer
4	0.1	chain fatty acid	113	chain fatty acid	1.33	dietary fiber
5	0.07	expression	99	expression	1.31	gut microbiota
6	0.07	dietary fiber	92	dietary fiber	1.23	celiac disease
7	0.07	nf kappa b	85	children	1.19	expression
8	0.07	celiac disease	81	coliti	1.19	irritable bowel syndrome
9	0.06	children	79	bowel disease	1.19	fish oil
10	0.06	disease	77	association	1.18	intestinal microbiota
11	0.06	colorectal cancer	76	nf kappa b	1.16	risk
12	0.06	irritable bowel syndrome	75	disease	1.14	disease
13	0.04	coliti	73	risk factor	1.14	fecal microbiota
14	0.04	bowel disease	72	colorectal cancer	1.13	prevalence
15	0.04	association	72	intestinal epithelial cell	1.12	pattern
