# Supplementary figures and images for: Effect of Dietary Patterns on Inflammatory Bowel Disease: A Machine Learning Bibliometric and Visualization Analysis
Source: Nutrients. 2023 Aug 3;15(15):3442. doi: 10.3390/nu15153442 (PMC10420952; doi:10.3390/nu15153442)

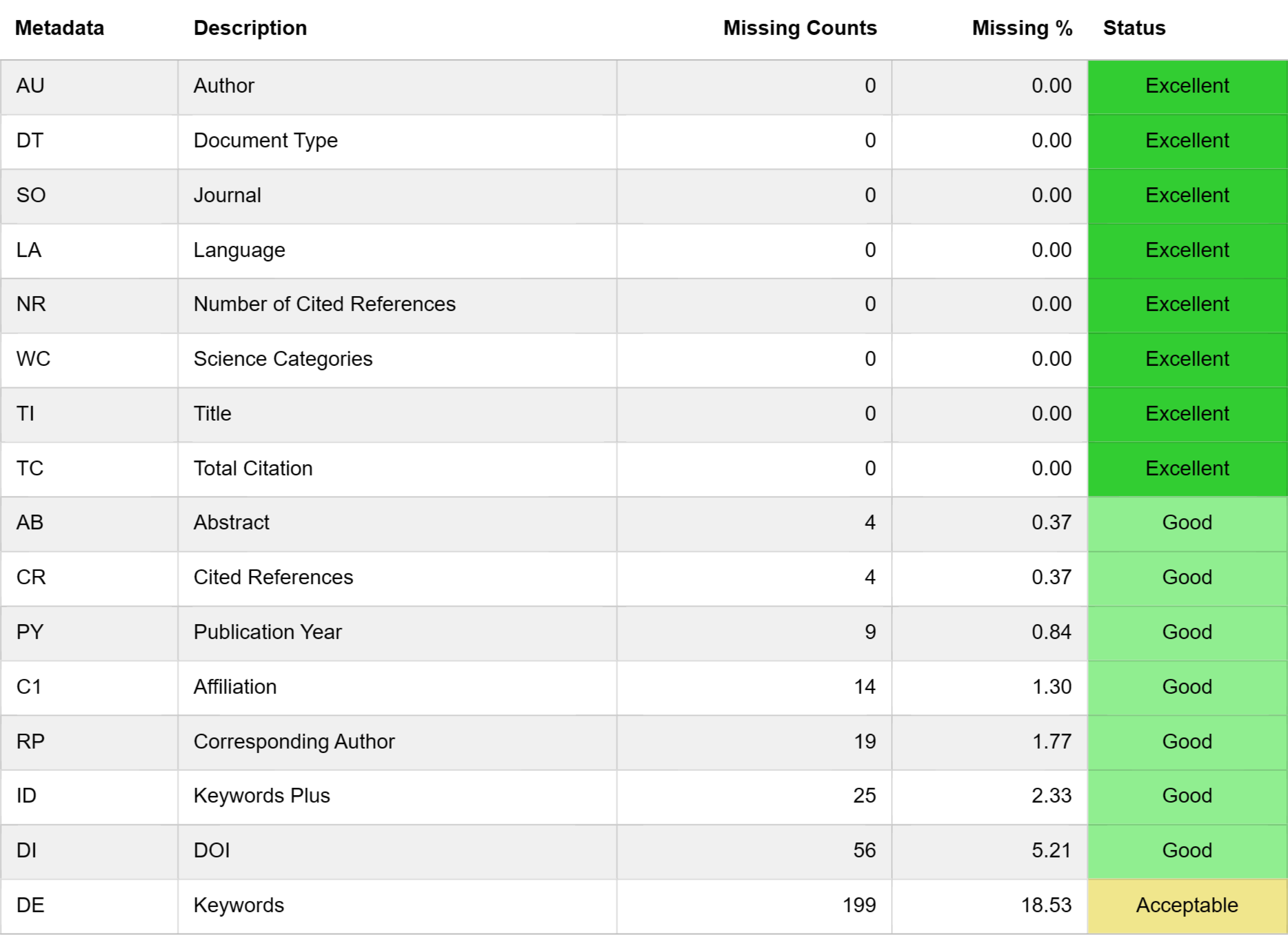

Supplement: Supplementary file 1 [file nutrients-15-03442-s001.zip › Supplementary Figure S1.tif]

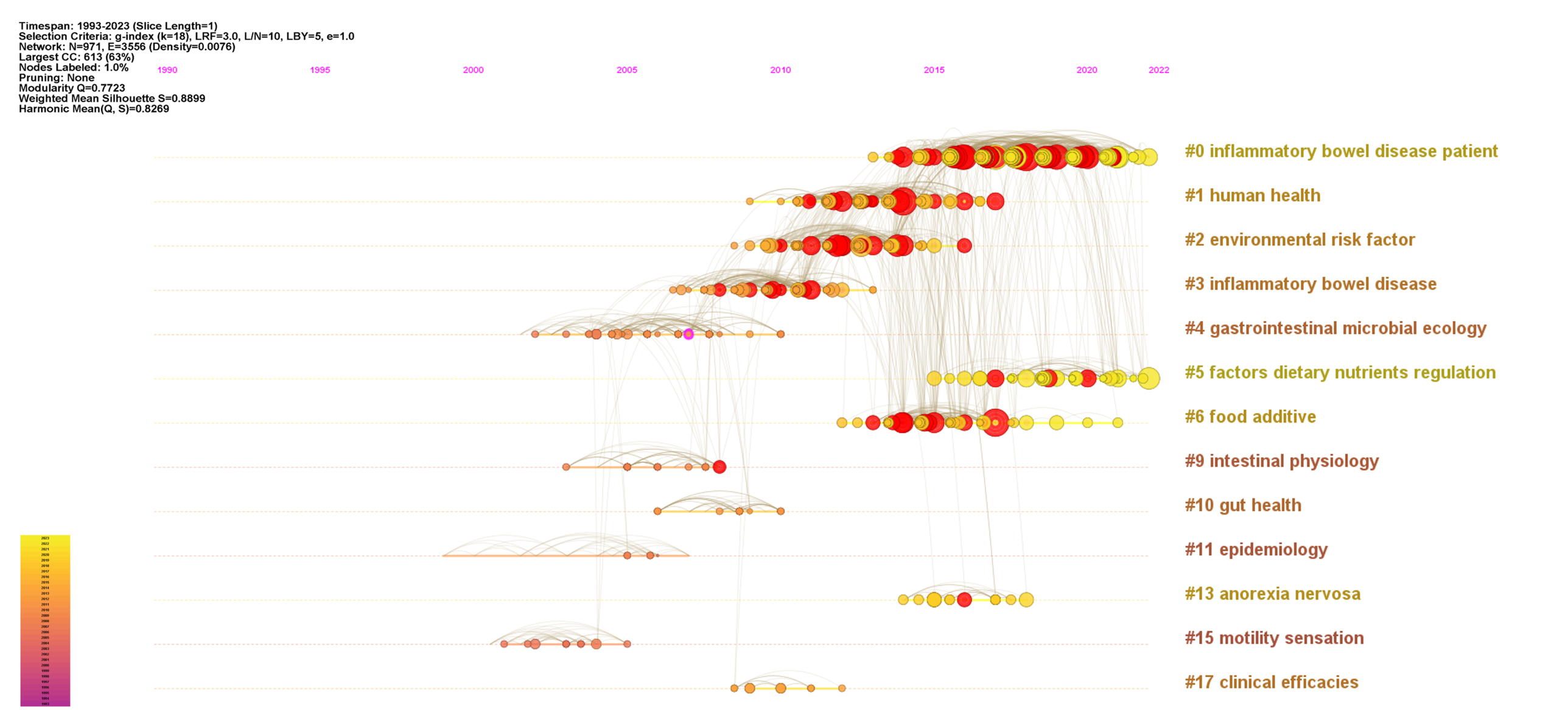

Supplement: Supplementary file 1 [file nutrients-15-03442-s001.zip › Supplementary Figure S3.tif]
